# Cross-sectional and longitudinal associations between depressive symptoms and cognitive performance in mild cognitive impairment

**DOI:** 10.1192/bjp.2025.10341

**Published:** 2025-08-27

**Authors:** Calum A Hamilton, Paul C Donaghy, John-Paul Taylor, Joanna Ciafone, Rory Durcan, Michael Firbank, Gemma Greenfinch, Louise M Allan, John T O’Brien, Alan J Thomas

**Affiliations:** 1 Translational and Clinical Research Institute, Newcastle University, United Kingdom; 2 Institute of Nuclear Medicine, University College London Hospitals, United Kingdom; 3 Centre for Research in Ageing and Cognitive Health, University of Exeter, United Kingdom; 4 Department of Psychiatry, School of Clinical Medicine, University of Cambridge, United Kingdom

**Keywords:** mild cognitive impairment, Alzheimer’s disease, dementia with Lewy bodies, depression

## Abstract

**Background:**

Depressive symptoms are common in mild cognitive impairment (MCI). These may be associated with poorer cognitive function, and increased risks of dementia transition.

**Aims:**

We aimed to examine the cognitive patterns associated with variations in depressive symptoms in neurodegenerative MCI without primary mood disorder.

**Method:**

Individuals with MCI (n=123) including MCI due to Alzheimer’s disease (n=54) and MCI with Lewy bodies (n=69) underwent repeated annual assessment of cognitive function and concurrent depressive symptoms using the Addenbrooke’s Cognitive Examination – Revised and Geriatric Depression Scale-15, respectively.

Between- and within-person differences in depressive symptoms were disaggregated and related to between- and within-person cognitive differences and modification of cognitive performance trajectories over time.

**Results:**

There was strong evidence of a state-based association between depressive symptoms and cognitive function. Intra-individual differences in depressive symptoms were negatively associated with concurrent cognitive performance such that a two-point increase in depressive score explained a one-point decrease in cognitive score, on average (Point Estimate = -0.56, 95% CrI = -1.05 to -0.08).

The data did not support a trait-based association between depressive symptoms and cognitive performance (Point Estimate = 0.10, 95% CrI = -0.42 to 0.59), nor any between- or within-person trajectory modification associated with depressive symptoms.

**Conclusions:**

Within-person variations in depressive symptom severity are associated with acute cognitive performance differences. Cognitive scores derived during active depressive periods may under-estimate longer-term cognitive capabilities. Treating depressive symptoms in MCI may clarify underlying cognitive performance capacity, and help maintain optimal cognitive function for longer.

## Background

Mild cognitive impairment (MCI) is an age-related condition primarily characterised by cognitive dysfunction, but with maintenance of independent function, differentiating this from dementia. MCI may be a prodromal stage of a developing dementia, as previously described in Alzheimer’s disease (AD) and dementia with Lewy bodies (DLB).

Depressive symptoms are a common feature of both MCI and dementia. Greater levels of depressive symptoms have been associated with poorer cognitive function in MCI,^1-3^ and greater risks of progression from MCI to dementia^4-6^ (though findings are mixed, with depressive symptoms also being associated with reversion from MCI to normal cognition^7^). Depressive symptoms may therefore be an indicator of the severity and prognosis of MCI.

However, the reasons for this are not clear. As in dementia,^8^ a cross-sectional relationship between depressive symptoms and cognitive function in MCI may reflect the aggregation of several effects, including both between- and within-person differences. Disaggregating and assessing these effects may provide clues as to the underlying factors which contribute to associations between depressiveness and cognitive impairment in MCI. These may be related to the pattern-based hypotheses that have been proposed to explain neurocognitive deficits in primary mood disorder, including ‘state’, ‘trait’, and ‘scar’ effects,^9^ or may reflect other factors specific to neurodegenerative conditions.

### Within-person state effect

**Figure 1:**
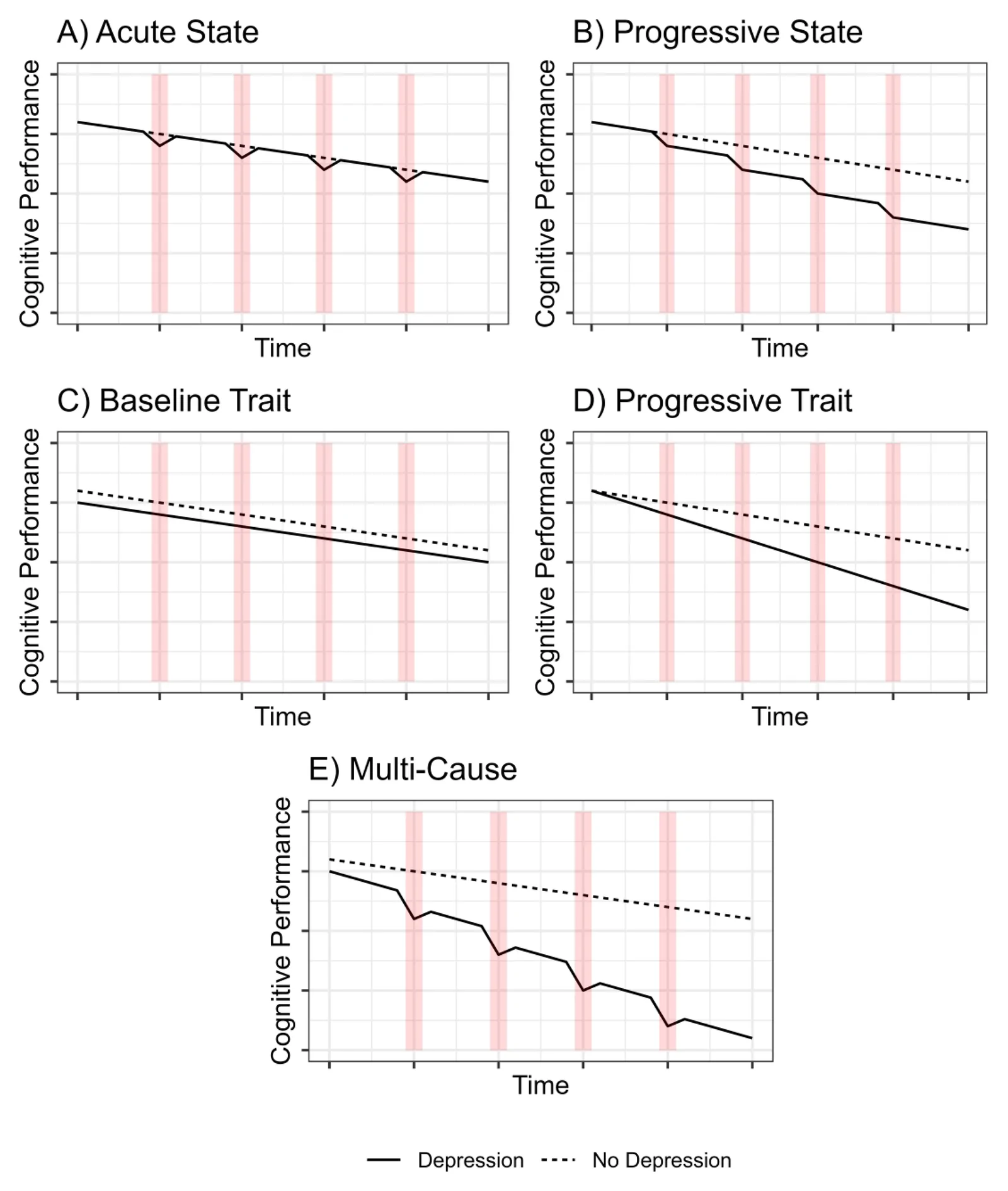
Theoretical models of cognitive performance for individuals with MCI and trait-level depressive symptoms (solid line) experiencing multiple transient depressive episodes (red bars), vs MCI without depressive symptoms (dashed line).

When assessed during the immediate depressed state, individuals may perform worse at cognitive tasks, with this deficit resolving with the remission of depressive symptoms (see Figure 1a). This ‘state’^9^ effect may reflect the acute influences of impaired concentration in depression leading to poorer cognitive performance. While this would not be a unique characteristic of MCI, individuals with a pre-existing neurodegenerative cognitive impairment may be particularly vulnerable to any further disruption to cognitive performance.

### Within-person trajectory modification

Within-person worsening of depressive symptoms may be associated with acceleration of the rate of cognitive decline (see Figure 1b). This could reflect the neurotoxic effects of depression (incorporating the ‘scar-based relationship’ previously described in neurocognitive development^10^ and adult major depressive disorder^9^), emergent depressiveness as a symptom of underlying disease progression, or a rise in depressive symptoms in response to recent decline in cognitive function.^11^

### Between-person trait effect

Individuals with a higher baseline for depressive symptoms may also typically have a lower cognitive baseline (see Figure 1c). This could reflect between-person differences in lifelong risk/protective factors which influence baseline levels of both cognitive function and depressiveness. Examples may include shared genetic influences, age, education, earlier life adversity, and persistent cognitive impairments in people with a history of major depression.^12^

### Between-person trajectory differences

Individuals with a higher depressive baseline may also experience a faster long-term cognitive decline (see Figure 1d). This could point to between-person differences in underlying neuropathological change as a possible shared origin for this relationship.^13^ Early and sustained depression may be a prodromal manifestation of underlying dementia-related neuropathology, emerging prior to or in parallel to MCI. Alternatively, long-term depression may worsen underlying pathophysiological changes in the brain, worsening rate of neurocognitive decline.

Importantly, these are not necessarily mutually exclusive effects: associations between depressive and cognitive symptoms over time could reflect the aggregation of multiple effects (see Figure 1e). While cross-sectional studies are limited in their ability to examine these different effects, longitudinal studies offer an opportunity to disaggregate between- and within-person differences in depressive symptoms,^14^ and relate these to time-varying and -invariant differences in cognitive performance.^15^

We therefore aimed to use repeated measurements of depressive and cognitive symptoms to simultaneously test the evidence for these four hypothesised effects in individuals at the prodromal stage of a neurodegenerative dementia.

## Methods

### Participants

Participants were drawn from two sequential studies of individuals with MCI, with recruitment, diagnosis and differential classifications for each previously described in detail.^16, 17^ Individuals aged 60 years or older with a health service diagnosis of MCI were screened from older person’s memory, psychiatry, neurology and other secondary/tertiary medical services in North East England (N=179). They were required to be free of dementia at baseline, with MCI diagnosis validated at screening. Presence of major depressive disorder was cause for exclusion at baseline.

Of those screened for inclusion, N=152 were considered eligible for retention at baseline. Analyses were restricted to those diagnosed with probable MCI with Lewy bodies or probable MCI due to Alzheimer’s disease.

All prospective participants gave written informed consent to participate.

### Design

Both cohort studies incorporated repeated annual longitudinal assessment of cognitive function, and also provided repeated annual measures of concurrent depressive symptoms. Repeated follow-up was discontinued after onset of dementia.

Up to ten years of follow-up were available at the point of data locking, though the average length of follow-up was 2.6 years, with dementia or death commonly occurring within 3 years.^18^ Participants had a median of 3 timepoints available for analysis (interquartile range = 2-4, minimum = 1, maximum = 8). Individuals lost to follow-up were retained for analysis with any available data included to provide additional information and reduce possible bias from their exclusion.

### Measures

Global cognitive function was assessed with the 100-item Addenbrooke’s Cognitive Examination – Revised (ACE-R) from which a standardised mini mental state examination (MMSE) equivalent score may be derived.

Depressive symptoms at each annual visit were assessed using the 15-item Geriatric Depression Scale (GDS-15), administered by the researcher. Participants were asked to respond to the GDS-15 questions with consideration of how they had been feeling over the past week. In the event that any question was missed, the equivalent 15-item score was prorated based on completed questions.

A subset of participants had informants available, who completed the Neuropsychiatric Inventory (NPI) questionnaire to provide additional contextual information.

### Diagnoses

Participants were differentially classified as MCI due to Alzheimer’s disease (MCI-AD) or MCI with Lewy bodies (MCI-LB) according to consensus clinical and research criteria,^19, 20^ respectively. Suspected vascular, frontotemporal, or other MCI aetiologies were excluded, as were those whose MCI was primarily attributable to major psychiatric illness, including major depression.

### Analysis

Associations between depressive and cognitive symptoms were assessed with a hierarchical Bayesian model, with ACE-R and MMSE included as the primary and secondary outcome measures respectively. In both cases, outcomes were adjusted for age and education.

Between- and within-subject differences in depressive symptoms were disaggregated by participant mean centring of time-varying GDS-15 values. Participant-level intercept and centred values were included as between- and within-person predictors of cognitive performance, respectively.

Analyses adjusted for age and education based on *a priori* reasoning that these would be important covariates for cognitive function, as well as adjusting for the underlying time trend to account for the effect of disease progression on cognitive function, and included between- and within-person time interactions to examine associations with progression rates. Sensitivity analyses also assessed any modifications induced by inclusion of diagnostic sub-group interactions, or daytime somnolence measured with the Epworth Sleepiness Scale (ESS).

We provide point estimates (β) of the posterior distribution as measures of the estimated strength of associations between unit-changes in depressive symptoms and ACE-R performance (i.e. β = 1 corresponds to a 1-point difference in the ACE-R), with uncertainty in these estimates quantified by Bayesian 95% Credible Intervals (CrIs), measuring the probability of a given effect with respect to the data to hand and the (weakly regularised) prior beliefs about the likelihood of a given effect size.

Weakly informative regularising priors codify the prior belief that small raw effect sizes (|β| < 1) are substantially more likely than large ones (|β| > 1), with a null effect (β = 0) the most likely for all tested associations.

Models were estimated using the *brms* package for R software as an interface to the *Stan* probabilistic programming language, using Hamiltonian Monte Carlo/No-U-Turn Sampler. Weakly informative zero-centred regularising priors were included for all hypotheses-testing parameters.

### Ethics and Data

Both cohort studies received favourable ethical approval from the NHS National Research Ethics Service Committee North East – Newcastle and North Tyneside 2 (12/NE/0290 and 15/NE/0420).

Data supporting this analysis are available upon request from the corresponding author, or via Dementias Platform UK.

## Results

### Summary measures

N=123 of the overall MCI cohort were available for this analysis, with a consensus baseline diagnosis of either probable MCI-LB (N=69) or MCI-AD (N=54). Participant study baseline characteristics are provided in Table 1.

The average absolute difference between GDS-15 scores on sequential retesting was 1.9 (SD = 1.41), with a maximum of 11, and considerable variation between these points (see Supplementary Figure S2B). There was no evidence of a greater retest variability in MCI-LB in comparison to MCI-AD (Point Estimate = -0.27, 95% CrI = -0.98 to 0.44).

Of the 123 MCI cases, 76 (62%) had an informant available to complete the Neuropsychiatric Inventory (NPI), with 46/76 (61%) reporting depression in the participant. Of these 46 with informant-reported depression, 40 informants (87%) reported being distressed due to the participant’s depressive symptoms, with 25 (54%) reporting moderate/severe/very severe distress.

### Between and within-subject associations

There was evidence of a negative within-person association between depressive symptoms and concurrent cognitive performance in the ACE-R (Point Estimate = - 0.56, 95% CrI = -1.05 to -0.08), supporting the hypothesised within-person state-based relationship (Figure 1A).

There was no evidence of a within-person association between depressive symptoms and rate of cognitive decline (Point Estimate = 0.01, 95% CrI = -0.18 to 0.19), which did not support the hypothesised within-person modification of the cognitive trajectory (Figure 1B).

There was no evidence of a between-person association between trait-level depressiveness and baseline cognitive performance in the ACE-R (Point Estimate = 0.10, 95% CrI = -0.42 to 0.59), which did not support the hypothesised between-person trait-based relationship (Figure 1C).

Finally, there was no evidence of a between-person association between depressiveness and rate of cognitive decline in the ACE-R (Point Estimate = 0.07, 95% CrI = -0.15 to 0.28), which did not support the hypothesised between-person differences in decline (Figure 1D).

**Figure 2:**
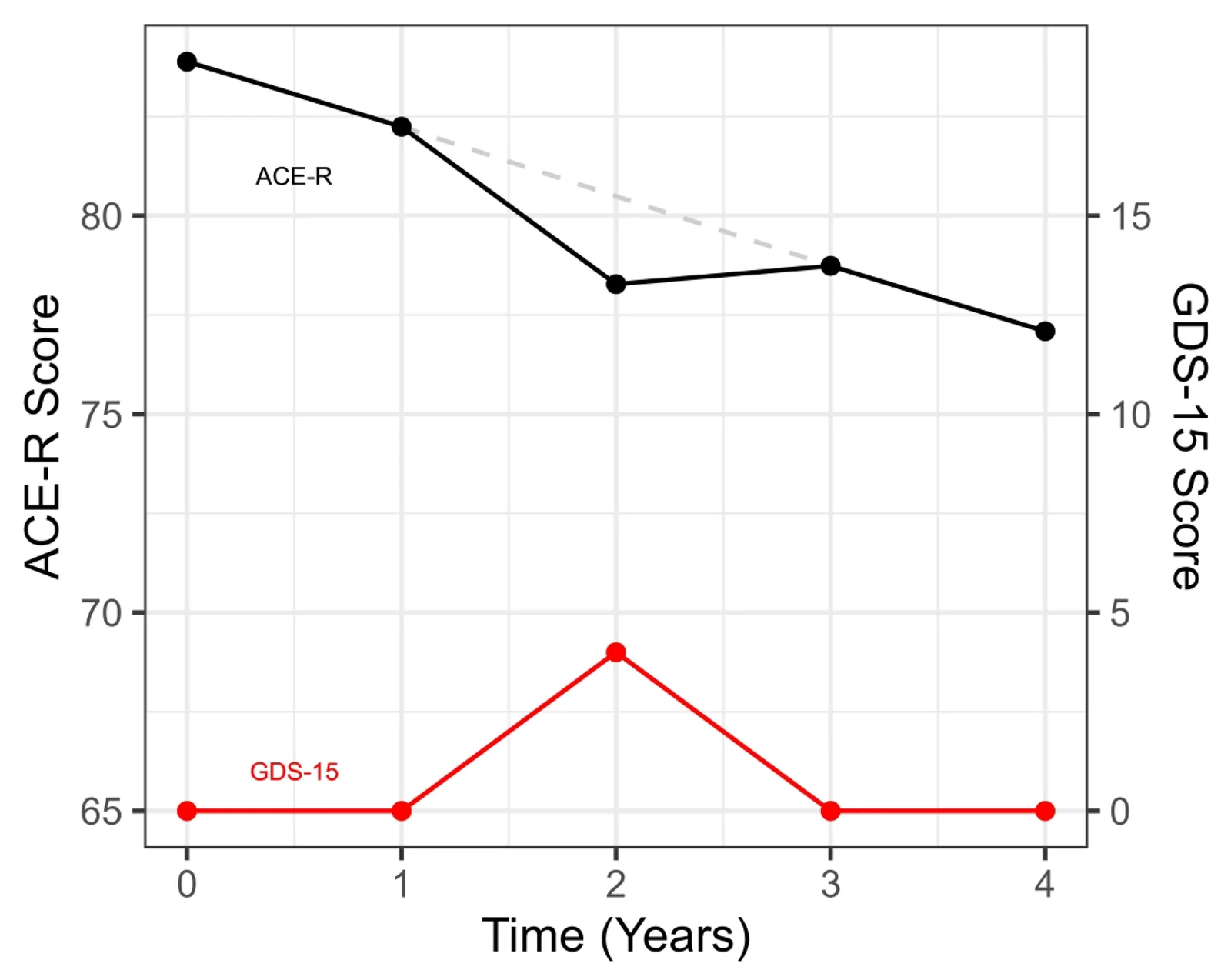
Model-predicted cognitive performance over time for a simulated MCI patient, showing an acute performance deficit coinciding with increased depressive symptoms but subsequent return to the underlying trajectory.

The data were therefore most compatible with a within-person association between cognitive and depressive symptoms manifesting as an acute deviation from the underlying cognitive trajectory in periods where individuals had more depressive symptoms, but without modification of the underlying trajectory of decline (see Figure 2).

The same state-based effect was observed for the secondary outcome MMSE, though with a smaller raw effect size (Point Estimate = -0.20, 95% CrI = -0.39 to - 0.01).

### Effect size contextualised

These results indicated that there was a high probability of a negative within-subject association between GDS-15 change and ACE-R scores, with a 2-point change in GDS-15 corresponding to a >1 point change in ACE-R score on average.

The estimated annual change in the ACE-R in this cohort was -2.1 points (95% CrI = -3.2 to -0.9) suggesting that, on average, a 2-point intra-individual difference in GDS-15 was equivalent to ~6 additional months of progressive decline on retesting.

### Reliability and sensitivity analyses

Despite greater depressiveness on average in MCI-LB compared to MCI-AD (see Table 1), a sensitivity analysis did not support any aetiology × depression interaction for the trait-level (Point Estimate = -0.04, 95% CrI = -0.73 to 0.64) or state-level (Point Estimate = 0.28, 95% CrI = -0.38 to 0.93) main effects. We also assessed these associations in a further sensitivity analysis in separate sub-groups. This identified posterior distributions of within-person depressive associations in both MCI-AD (β = -0.69, 95% CrI = -1.23 to -0.13) and MCI-LB (β = -0.35, 95% CrI = -0.83 to 0.11) which were consistent with the effect identified in the primary analysis, though less certain in MCI-LB in particular.

In a sensitivity analysis, we estimated an additional multivariate outcome model to assess the associations of concurrent depressiveness with performance in each ACE-R sub-domain, adjusting for MCI sub-group differences in cognitive profile and residual correlations between sub-domain scores. This indicated that the associations of concurrent depressiveness were primarily manifest in the Memory (β = -0.32, 95% CrI = -0.56 to -0.08), Verbal Fluency (β = -0.14, 95% CrI = -0.29 to 0.01), and Attention/Orientation (β = -0.09, 95% CrI = -0.21 to 0.02) sub-domains.

To assess whether this relationship might be explained by poorer quality of sleep at night, and consequently greater daytime somnolence, we conducted an additional sensitivity analysis incorporating time-varying Epworth Sleepiness Scale scores. This did not meaningfully change the results, and ESS scores did not evidently account for any additional variation in cognitive performance.

All sampling chains showed good convergence with sufficient effective sample size. Posterior distributions and Hamiltonian Monte Carlo trace plots for key parameters are presented in Supplementary Figure S1.

## Discussion

We hypothesised that depressive symptoms would be associated with poorer cognitive performance and/or more severe rate of decline in MCI, reflecting the possible aggregation of several between- and within-person effects.

We found evidence that within-person variations in depressive symptoms were associated with acute variations in cognitive performance, supporting the hypothesised within-person state effect. However, we did not find any clear evidence of a modification of the trajectory of decline attributable to within- or between-person differences in depressive symptoms, nor any trait-level associations.

These data were therefore most consistent with a state-based association between depressive and cognitive symptoms in MCI. These findings are consistent with previous literature observing an acute impairment of cognitive performance in periods of depression in adolescents^21, 22^, adults^23^, and older adults.^24^ In the short term, these performance deficits may exaggerate or mimic annual decline. This emphasises a need for caution when interpreting changes in cognitive test performance when depressive symptoms are also present.

This cognitive performance deficit associated with concurrent depressive symptoms could give the impression of faster decline, explaining increased risks of MCI to dementia transitions found in some studies.^6^ Improvements in depressive symptoms could conversely lead to an apparent resolution of cognitive deficits, thereby accounting for the associated reversion of MCI to healthy cognition reported in other studies.^7^

Differences between these findings and those of some previous studies may reflect some important aspects of this study design and analysis. Depressive symptoms are commonly assessed at study baseline only, and consequently treated as time-invariant. A single measure of depressive symptom severity represents the aggregation of several between- and within-person effects. While previous studies have incorporated time-varying measures of depressive symptoms, they do not typically disaggregate between-from within-person differences. This emphasises the potential value of this analytical approach when repeated measures are available.

Depressive symptoms were measured in a probable neurodegenerative population where a current episode of major depression was a cause for exclusion and so by definition they typically have low levels of depressive symptoms which are unlikely to be treated in clinical practice, with average baseline GDS-15 score in this cohort being below the threshold of typical normal mood/mild depressiveness cut-offs. Other cohorts have examined the cognitive outcomes of clinically diagnosed depressive disorders (e.g. major depressive disorder or bipolar depression), or cognitive, clinical or pathological outcomes in MCI with comorbid clinical depression. Between- and within-person differences in GDS-15 scores may therefore largely reflect fluctuations in sub-clinical depressive symptoms which may not have the same long-term implications as the pronounced and sustained depressive symptoms in primary mood disorders. Nevertheless, 87% of caregivers in this study reported distress associated with these symptoms, most of these being moderate-to-severely distressed, and we have demonstrated that these common depressive symptoms are also associated with cognitive performance. This suggests that even mild depressive symptoms may contribute to distress and cognitive outcomes.

These findings reinforce the relevance of depressive symptoms to the interpretation of cognitive change in MCI in clinical or research settings. Within-person fluctuations in depressive symptoms may obscure the true cognitive trajectory over the course of follow-up in MCI. An increase in depressive symptoms at follow-up could mimic or exaggerate cognitive decline. Conversely, a reduction in depressive symptoms at follow-up could attenuate or mask a true decline in cognitive function.

In clinical settings, any change in cognitive scores at follow-up will need to be interpreted with consideration of concurrent changes in depressive symptoms. We demonstrate here that within-person fluctuations in depressive symptom severity could be associated with a measurable change on standardised cognitive tests such as the ACE-R even at sub-clinical levels of depressiveness. A 2-point intra-individual difference in GDS-15 score (the average absolute difference on sequential retesting, though many individuals showed substantially greater variability than this two-point average) was sufficient to account for a 1-point ACE-R difference on average, equivalent to an additional 6-months of progression in this cohort. This small association may not translate into overt differences in clinical course (i.e. conversion from MCI to dementia), but may account for some of the test-retest variation within individuals, alongside other well-understood between-person sources of variation such as age and education. As this did not clearly modify the underlying trajectory however, reduction of depressive symptoms could therefore help to maintain optimal cognitive performance for longer, with time saved comparable to emerging disease-modifying therapies.^25^

This effect was also seen, though less overt, when using the less granular MMSE. These findings suggest that there is likely to be a measurable impact on cognitive screening tests such as these in individuals with MCI. However, our posterior distributions will require updating in external cohorts to provide more precise estimates of the size of this effect.

These findings highlight that the most evident effect in this cohort is the state-based depressive effect. However, other explanations continue to be relevant more widely, with this cohort potentially being underpowered to detect more subtle scar-like or trait-based cognitive effects, or having insufficiently severe history of depressive symptoms to detect longer-term effects.

Sub-group analyses supported similar associations in both MCI-AD and MCI-LB, suggesting that this was not an association specific to either one of the two MCI sub-groups, and consequently aetiology-nonspecific. There are several possible explanations for this apparent state-based, disease-nonspecific association, some of which we were able to investigate in sensitivity analyses: specifically, impaired attention and concentration specifically, and poorer sleep quality in more depressed periods.

Sensitivity analyses indicated that concurrent depressiveness was most likely associated with poorer performance in memory, verbal fluency, and attention/orientation domains of the ACE-R. Previous analysis of a subset of this cohort with comprehensive neuropsychological assessment at baseline indicated that over 60% of group-based variance in verbal learning and memory performance in both MCI-LB and MCI-AD were explained by slowing of processing speed.^26^ These higher-order deficits associated with depressiveness may therefore reflect associated slowing of processing speed, though we did not have this detailed neuropsychological assessment available at repeated follow-up to test this empirically, highlighting a need for further detailed investigation. We also assessed whether daytime somnolence might mediate this association, but including this as a time-varying covariate did not clearly influence the results.

Aside from motivation, attention, and sleep related effects, this association could be explained by shared neurobiological factors. While this time-specific effect may be inconsistent with longer-term structural brain changes associated with acute low mood, this could alternatively be explained by acute functional abnormalities underlying concurrent periods of both low mood and lower cognitive performance,^27^ though we were unable to assess this using the available data.

This work has several limitations to consider. Diagnoses of AD were made according to clinical characteristics, without confirmatory biomarkers, leaving their exact aetiology uncertain. However, participants were approached for brain tissue donation, and to date, 100% of the AD classifications (4/4) were validated at autopsy.

As a suspected neurodegenerative sample, our MCI cohort were inherently at high risk of loss to follow-up for several reasons, including drop-out due to illness, onset of dementia, or death. As a result, these findings were drawn from a relatively brief window of follow-up time. These findings will require replication in both larger and longer studies. However, the brief window of time between onset of a neurodegenerative MCI and transition to a more severe clinical state may impose an upper limit on the overall duration of follow-up feasible for most MCI participants. This may be circumvented through more frequent re-assessment of cognitive and depressive symptoms (e.g. monthly rather than annual), potentially leveraging remote digital cognitive/mood assessments, to increase the number of repeated observations within the relatively brief follow-up period available.

## Conclusions

Quantifiable associations between cognitive performance and depressive symptoms in MCI were largely isolated to an acute state-based effect. In clinical settings, variations in cognitive performance in MCI should be considered within the wider context of any concurrent changes in depressive symptoms. Addressing depressive symptoms in MCI may have a secondary effect of remediating some cognitive symptoms, though this requires further investigation into the causal direction and any possible shared underlying causes of this likely multi-faceted relationship.

## Supplementary Material

Supplementary Materials

## Figures and Tables

**Table 1:** Baseline characteristics of the sample.

	Overall, N = 123^*1*^	MCI-AD, N = 54^*1*^	Probable MCI-LB, N = 69^*1*^
ACE-R Global Score	80.7 (9.3)	80.9 (9.8)	80.6 (8.9)
Age	75.6 (7.2)	76.2 (7.6)	75.1 (6.8)
Education	11.9 (2.9)	12.4 (3.3)	11.5 (2.6)
Female Gender	48 (39%)	33 (61%)	15 (22%)
15-Item Geriatric Depression Scale	4.0 (3.5)	3.2 (2.7)	4.7 (3.9)

1 Mean (SD); n (%)

## Data Availability

Data from the cohorts used in these analyses are available through the Dementias Platform UK data portal.

## References

[1] Johnson LA, Mauer C, Jahn D (2013). Cognitive differences among depressed and non‐depressed MCI participants: A project FRONTIER study. International journal of geriatric psychiatry.

[2] Yoon S, Shin C, Han C (2017). Depression and cognitive function in mild cognitive impairment: a 1-year follow-up study. Journal of geriatric psychiatry and neurology.

[3] Zahodne LB, Tremont G (2013). Unique effects of apathy and depression signs on cognition and function in amnestic mild cognitive impairment. International journal of geriatric psychiatry.

[4] Modrego PJ, Ferrández J (2004). Depression in patients with mild cognitive impairment increases the risk of developing dementia of Alzheimer type: a prospective cohort study. Arch Neurol.

[5] Lee GJ, Lu PH, Hua X (2012). Depressive symptoms in mild cognitive impairment predict greater atrophy in Alzheimer’s disease-related regions. Biol Psychiatry.

[6] Qin Y, Han H, Li Y (2023). Estimating Bidirectional Transitions and Identifying Predictors of Mild Cognitive Impairment. Neurology.

[7] Sanz-Blasco R, Ruiz-Sánchez de León JM, Ávila-Villanueva M, Valentí-Soler M, Gómez-Ramírez J, Fernández-Blázquez MA (2022). Transition from mild cognitive impairment to normal cognition: Determining the predictors of reversion with multi-state Markov models. Alzheimer’s & Dementia.

[8] Bennett S, Thomas AJ (2014). Depression and dementia: cause, consequence or coincidence?. Maturitas.

[9] Hammar Å, Ronold EH, Rekkedal G (2022). Cognitive Impairment and Neurocognitive Profiles in Major Depression-A Clinical Perspective. Front Psychiatry.

[10] Allott K, Fisher CA, Amminger GP, Goodall J, Hetrick S (2016). Characterizing neurocognitive impairment in young people with major depression: state, trait, or scar?. Brain Behav.

[11] Jajodia A, Borders A (2011). Memory predicts changes in depressive symptoms in older adults: a bidirectional longitudinal analysis. J Gerontol B Psychol Sci Soc Sci.

[12] Bora E, Harrison BJ, Yücel M, Pantelis C (2013). Cognitive impairment in euthymic major depressive disorder: a meta-analysis. Psychol Med.

[13] Khundakar AA, Thomas AJ (2015). Neuropathology of depression in Alzheimer’s disease: current knowledge and the potential for new treatments. J Alzheimers Dis.

[14] Curran PJ, Lee T, Howard AL, Lane S, MacCallum R (2012). Advances in longitudinal methods in the social and behavioral sciences.

[15] Graziane JA, Beer JC, Snitz BE, Chang C-CH, Ganguli M (2016). Dual Trajectories of Depression and Cognition: A Longitudinal Population-Based Study. The American Journal of Geriatric Psychiatry.

[16] Donaghy PC, Ciafone J, Durcan R (2022). Mild cognitive impairment with Lewy bodies: neuropsychiatric supportive symptoms and cognitive profile. Psychol Med.

[17] Donaghy PC, Taylor JP, O’Brien JT (2018). Neuropsychiatric symptoms and cognitive profile in mild cognitive impairment with Lewy bodies. Psychol Med.

[18] Hamilton CA, Donaghy PC, Durcan R (2024). Outcomes of Patients With Mild Cognitive Impairment With Lewy Bodies or Alzheimer Disease at 3 and 5 Years After Diagnosis. Neurology.

[19] Albert MS, DeKosky ST, Dickson D (2011). The diagnosis of mild cognitive impairment due to Alzheimer’s disease: Recommendations from the National Institute on Aging-Alzheimer’s Association workgroups on diagnostic guidelines for Alzheimer’s disease. Alzheimer’s & Dementia.

[20] McKeith IG, Ferman TJ, Thomas AJ (2020). Research criteria for the diagnosis of prodromal dementia with Lewy bodies. Neurology.

[21] Maalouf FT, Brent D, Clark L (2011). Neurocognitive impairment in adolescent major depressive disorder: State vs. trait illness markers. Journal of Affective Disorders.

[22] Bloch Yuval, Aviram Shai, Braw Yoram, Gvirts Hila Z, Rabany Liron, Walter Garry (2015). Attention Improves After Clinical Improvement in Acutely Depressed Adolescents. The Journal of Neuropsychiatry and Clinical Neurosciences.

[23] Gallagher P, Robinson LJ, Gray JM, Young AH, Porter RJ (2007). Neurocognitive Function Following Remission in Major Depressive Disorder: Potential Objective Marker of Response?. Australian & New Zealand Journal of Psychiatry.

[24] Köhler S, Thomas AJ, Barnett NA, O’Brien JT (2010). The pattern and course of cognitive impairment in late-life depression. Psychological medicine.

[25] Dickson SP, Wessels AM, Dowsett SA (2023). ‘Time Saved’ As a Demonstration of Clinical Meaningfulness and Illustrated Using the Donanemab TRAILBLAZER-ALZ Study Findings. The Journal of Prevention of Alzheimer’s Disease.

[26] Ciafone J, Thomas A, Durcan R (2021). Neuropsychological Impairments and Their Cognitive Architecture in Mild Cognitive Impairment (MCI) with Lewy Bodies and MCI-Alzheimer’s Disease. Journal of the International Neuropsychological Society.

[27] Miskowiak KW, Petersen CS (2019). Neuronal underpinnings of cognitive impairment and - improvement in mood disorders. CNS Spectr.

