# Supplementary material for: Cross-sectional and longitudinal associations between depressive symptoms and cognitive performance in mild cognitive impairment
Source: Br J Psychiatry. Author manuscript; Available in PMC 2025 Sep 9. (PMC7618092; doi:10.1192/bjp.2025.10341)
Supplement: Supplementary Materials [file EMS206860-supplement-Supplementary_Materials.pdf]

Supplementary Figure S1. Prior (orange) and posterior (blue) distributions for key hypotheses and corresponding MCMC sampling trace plots.

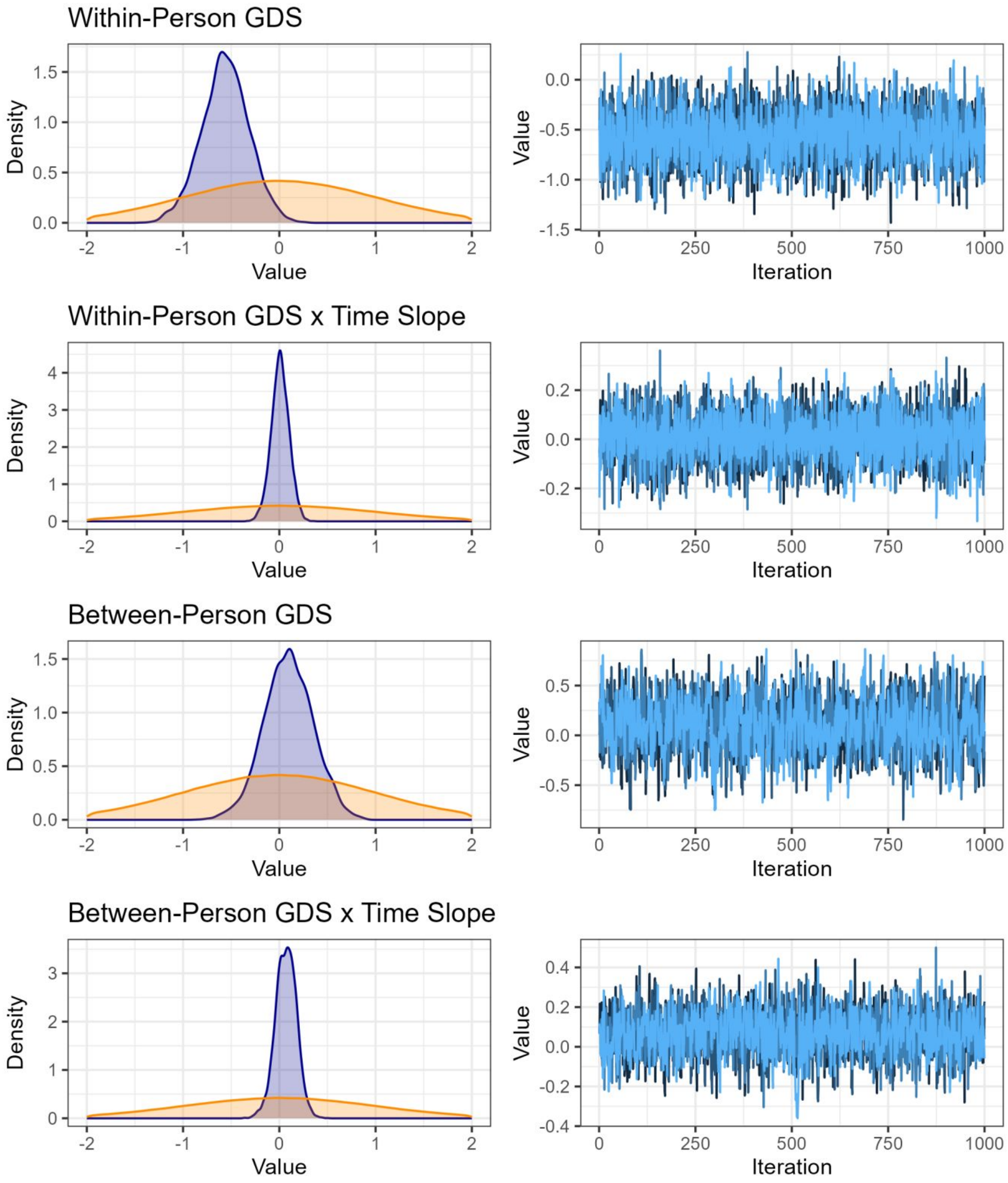

Supplementary Figure S2. Distributions of raw values and resulting residuals from primary

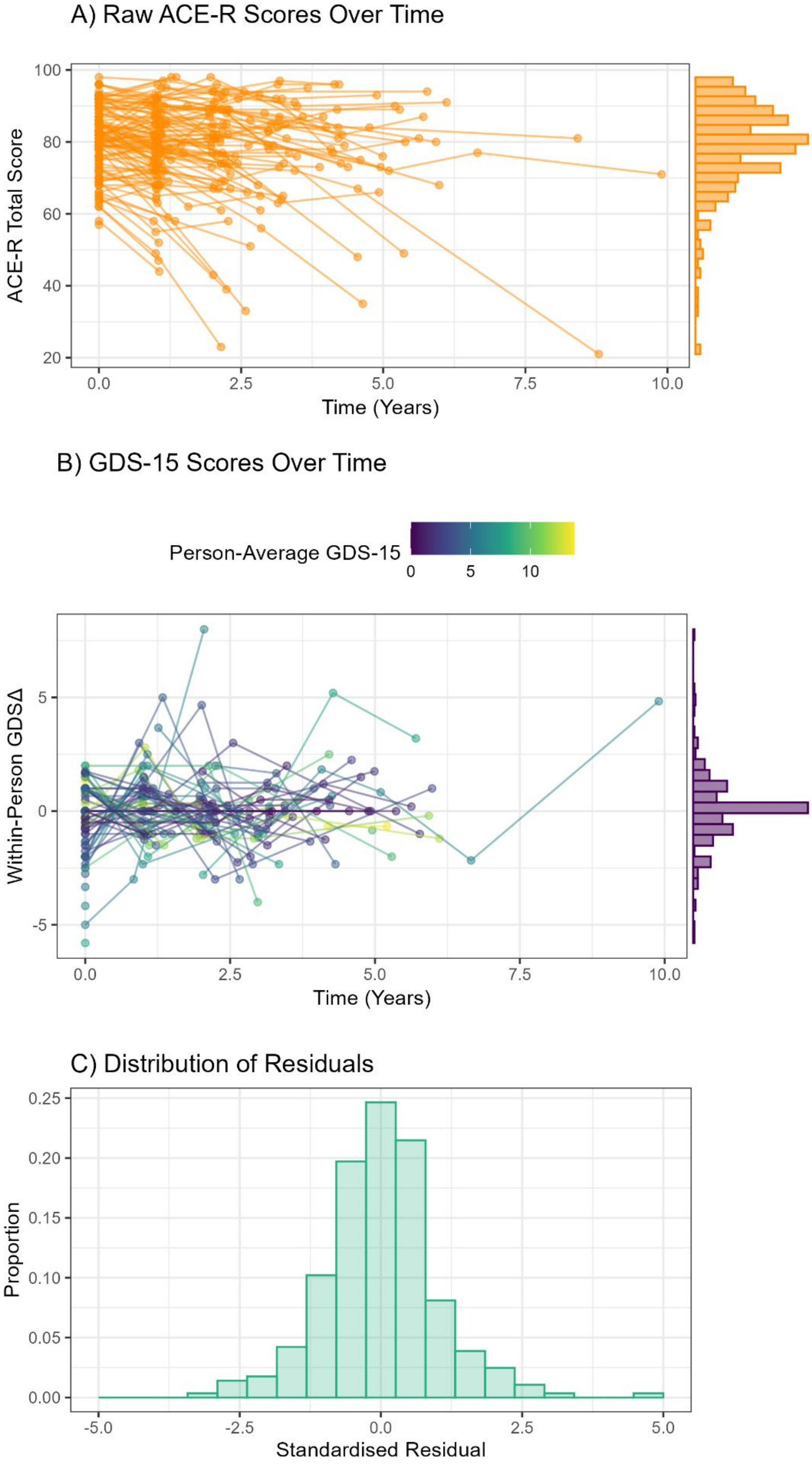

analysis.

Supplementary Analytical Code for Bayesian and Frequentist Models in R

```
###### supplementary model code #####

#bayesian model formulation reported in manuscript
bayes_model <- brm(ACER ~ 0 + Intercept +
                    #between person differences & allow for possible trajectory modification
                    Participant_Mean_GDS + Participant_Mean_GDS:time +
                    #within person differences & allow for possible trajectory modification
                    I(GDS - Participant_Mean_GDS) + I(GDS - Participant_Mean_GDS):Time +
                    #covariates
                    Time + I(Age-75) + I(Edu-11) +
                    #random effects
                    (Time | ID),
                   data = ...,
                   prior = c(#weakly informative half-normal prior for ACE-R SD
                             prior(normal(0, 10), class = sd),
                             #regularising zero-centered prior for all fixed effect coefficients
                             prior(normal(0, 1), class = b),
                             #weakly informative prior for expected baseline ACE-R in MCI
                             prior(normal(80, 10), class = b, coef = Intercept))

#frequentist model equivalent to above model estimated with flat priors
freq_model <- lmer(ACER ~ 1 +
                    #between person differences & allow for possible trajectory modification
                    Participant_Mean_GDS + Participant_Mean_GDS:time +
                    #within person differences & allow for possible trajectory modification
                    I(GDS - Participant_Mean_GDS) + I(GDS - Participant_Mean_GDS):Time +
                    #covariates
                    Time + I(Age-75) + I(Edu-11) +
                    #random effects
                    (Time | ID),
                   data = ...)
```
